# Incorporating clinical and demographic data into the Elixhauser Comorbidity Model: deriving and validating an enhanced model in a tertiary hospital’s internal medicine department

**DOI:** 10.1186/s12913-024-11663-z

**Published:** 2024-12-05

**Authors:** Gideon Leibner, David E. Katz, Yaakov Esayag, Nechama Kaufman, Shuli Brammli-Greenberg, Adam J. Rose

**Affiliations:** 1 Faculty of Medicine, Hebrew University of Jerusalem, P.O box 182, Beit-Horon, Jerusalem, 9093500 Israel; 2 Department of Internal Medicine, Shaare Zedek Medical Center, Jerusalem, Israel; 3 Department of Quality and Patient Safety, Shaare Zedek Medical Center, Jerusalem, Israel; 4 Department of Emergency Medicine, Shaare Zedek Medical Center, Jerusalem, Israel

**Keywords:** Risk adjustment, Prediction models, Illness severity, Case mix adjustment

## Abstract

**Background and objectives:**

The Elixhauser Comorbidity Model is a prominent, freely-available risk adjustment model which performs well in predicting outcomes of inpatient care. However, because it relies solely on diagnosis codes, it may not capture the full extent of patient complexity. Our objective was to enhance and validatethe Elixhauser Model by incorporating additional clinical and demographic data to improve the accuracy of outcome prediction.

**Methods:**

This retrospective observational cohort study included 55,945 admissions to the internal medicine service of a large tertiary care hospital in Jerusalem. A model was derived and validated to predict four primary outcomes. The four primary outcomes measured were length of stay (LOS), in-hospital mortality, readmission within 30 days, and increased care.

**Results:**

Initially, the Elixhauser Model was applied using standard Elixhauser definitions based on diagnosis codes. Subsequently, clinical variables such as laboratory test results, vital signs, and demographic information were added to the model. The expanded models demonstrated improved prediction compared to the baseline model. For example, the R^2^ for log LOS improved from 0.101 to 0.281 and the c-statistic to predict in-hospital mortality improved from 0.711 to 0.879.

**Conclusions:**

Adding readily available clinical and demographic data to the base Elixhauser model improves outcome prediction by a considerable margin. This enhanced model provides a more comprehensive representation of patients’ health status. It could be utilized to support decisions regarding admission and to what setting, determine suitability for home hospitalization, and facilitate differential payment adjustments based on patient complexity.

**Supplementary Information:**

The online version contains supplementary material available at 10.1186/s12913-024-11663-z.

## Background

Risk adjustment models are referred to as “*the use of information to calculate the expected health expenditures of individual… to improve efficiency and equity*“ [1, 2]. These models usually give a numerical score designed to quantify hospitalizaion outcomes and expected expenses based on patient data. They can form the basis for differential payment, which is the payment variation between individuals depending on their “adjusted risk”. These models can also be used to guide clinical [3], adjust for case mix as part of research studies, and to support health services planning and financing [2]. A variety of indexes are used for risk adjustment, including the Charlson Comorbidity Index (CCI) [4] and the Elixhauser Comorbidity Measure (ECM) [5]. While both the CCI and the ECM are based on a patient’s comorbid conditions, a number of studies have found the ECM to be superior to the CCI in terms of predicting adverse outcomes and cost [6–11].

However, despite their widespread acceptance and use, these models rely exclusively on comorbid illnesses, and thus may not fully capture a patient’s medical complexity. Few if any studies have attempted to expand the ECM model, incorporating broader clinical information to improve the prediction of patient outcomes [12–16].

Therefore, our objective in this study was to validate and examine how much the addition of clinical and demographic variables, such as vital signs and laboratory tests, as well as assigning weights to these different variables, adds to the ability of the ECM to predict four hospitalization outcomes: length of stay (LOS), in-hospital mortality, need for increased care (such as ICU), and 30-day readmission. The enhanced model could be used for differential payment by complexity, adjustment for illness severity in research studies, and to guide decisions about whom to admit and to what setting.

## Methods

### Database

In this retrospective observational cohort study, we used data from Shaare Zedek Medical Center (SZMC), a large tertiary care referral hospital in Jerusalem, Israel that serves a varied population in terms of ethnicity and socioeconomic status. We included patients who received care on the Internal Medicine service, either for all or part of their hospital stay. The study included 56,001 hospitalizations between 01.01.2016–12.31.2019. These dates were chosen to allow us to study care under usual conditions, prior to COVID-19. The study unit was hospitalizations rather than patients; therefore, some patients were included more than once. This approach is crucial in health systems risk adjustment, especially in internal medicine wards, where many patients are complex and have multiple hospitalizations. By considering each hospitalization as a separate unit, we can better standardize patient complexity and avoid losing important information, such as mortality events that occur during a specific hospitalization. Additionally, using hospitalizations better reflects the real-world scenario this model aims to simulate.

However, using hospitalization as the unit of observation introduces its own challenges and complexities, such as violating the assumption of independent observations due to multiple, and the potential for selection bias, as only survivors can accrue multiple admissions. To address these concerns, a sensitivity analysis was conducted to assess how the model’s performance might change if the last hospitalization of each patient was used as the unit of analysis. The results indicated only minor changes in model performance, as reflected in the C statistic (see supplementary file, *eFigure1*). Because we aimed to include all patients hospitalized in the internal medicine wards, the sole exclusion criterion implemented was age; specifically, patients under the age of 18 were excluded. Consequently, 56 patients who were hospitalized in the internal department for various reasons but did not meet this age criterion were excluded from our analysis, leaving a total of 55,945 observations.

To account for the variability in hospitalization durations we treated any length of stay less than one day as a full day in our analysis. Additionally, we applied a log transformation to the length of stay variable to address its right-skewed nature. In a sensitivity analysis, we also excluded hospitalizations longer than 150 days to assess their impact on the model’s performance. The results were only minimally changed, as shown in the supplementary file (*eFigure2).*

For the purposes of this study, “internal medicine wards” were defined as the four formal internal medicine wards (A, B, C, and D), geriatrics, cardiology, and the observation/short stay unit of the emergency room. Some Israeli hospitals do not have such units, and therefore the patients hospitalized at SZMC in these wards would be part of the population served by internal medicine in a different hospital setting. This study was approved by the research ethics committee of SZMC. (Approval number - SZ-0361-21).

Patients’ deidentified medical information was extracted from the hospital’s electronic medical record and included demographic characteristics, diagnosis codes, dates of service, and clinical data, including (but not limited to) laboratory results and vital signs. All independent variables were characterized based on their initial documentation, either upon emergency department arrival or later during admission to the department, whereas the dependent variables (outcomes) occurred subsequently.

### Dependent variables: patient outcomes

There were four dependent variables for this study. The first, which was continuous, was the length of stay (LOS), measured in days. Because LOS is not normally distributed, we performed a log transformation, which resulted in a normal distribution. As such, we report outcomes for the log-LOS [17]. The other three were binary outcomes: (1) in-hospital mortality; (2) hospital readmission within 30 days of discharge; (3) the need for increased care. Regarding readmissions, since our data is limited to SZMC, readmissions to other hospitals would not be captured, but SZMC patients are primarily readmitted back to SZMC [18].

Our definition of increased care was expansive and included any patient who spent part of their hospital stay in the Intensive Care Unit (ICU) or the Intermediate-Care Unit, which is a monitored setting within the IM ward, but with a lower patient to nurse ratio. It also included the receipt of mechanical ventilation, daytime bi-level positive pressure ventilation (BiPAP), or vasopressors (medications intended to support blood pressure) – even when they were provided in a regular ward bed. Daytime BiPAP was defined as occurring between 8 AM-8 PM. BiPAP received at night may be given for obstructive sleep apnea, but BiPAP received during the day is presumably intended as a substitute for mechanical ventilation. eMethods1 in the supplement contains a list of all vasopressors. Any of these interventions (i.e., ICU, Intermediate-Care, mechanical ventilation, daytime BiPAP, or vasopressors) sufficed to show that the patient had at least some degree of critical illness and required intensive intervention [19]. 

### Independent variables: elixhauser index & additional data

The basis of our model is the Elixhauser Index. The original Elixhauser paper defined 30 comorbid conditions, using the International Classification of Diseases, Version 9 (ICD-9). ICD-9 was used in developing our model over more updated systems since it is the coding system employed at our hospital. However, it is also possible to develop the extended model by adjusting the Elixhauser groups to use ICD-10 [20, 21]. We added additional variables to the model to better predict patient hospitalization outcomes. We considered many additional variables, first one at a time, and later in combination. The variables added to the ECM and ultimately retained in the model included:

Age; Sex; Elective Admission (1/0); Time Since Last Hospitalization (days); Prior Hospitalization LOS (days); Pulse (beats per minute); Systolic Blood Pressure (mmHg); Temp (Celsius); Pulse Oximetry (%); Creatinine (mg/dL); Albumin (g/dL); Hematocrit (%); C-reactive protein (CRP) (mg/dL); Serum Sodium (mEq/L); Serum Potassium (mEq/L); Serum Glucose (mg/dL); Norton-Scale – (a 4–20 scale, composed of five 1–4 subscales, namely physical condition; mental-condition; activity; mobility; incontinence).

Most of the variables above (e.g., vital signs, laboratory values) are self-evident; here we will briefly explain those that are less self-evident. The Norton Scale, which is rated subjectively by the admitting nurse, was developed in 1960 to help asses patients’ risk of developing a pressure ulcer [22]. The rationale behind the inclusion of this index in the model is that it serves as an indicator of a patient’s overall condition and mobility, which aligns with its utilization at SZMC. However, comparable indices for pressure ulcer risk, like the Braden scale [23], would likely yield similar advantages. Elective admissions directly to internal medicine are extremely uncommon; therefore, this variable was really capturing patients initially admitted to another service for an elective procedure, who were eventually transferred to internal medicine. All variables were captured as of the time of arrival to the hospital or to the medical ward. Some variables, such as albumin, were most often measured upon arrival to the ward rather than in the ED.

All continuous variables were transformed into categorical variables, with reference groups set as the normal state or the less risky state. In order to avoid removing observations with missing values from the final regressions, all missing variables were given the reference values. In many cases, this choice reflected the high likelihood that if a patient did not have a parameter measured (for example, serum albumin), it was probably because the treating clinician either suspected that the value would be normal or that it would not immediately impact the clinical management. This approach maintains predictive accuracy similar to models run without the variable that had a considerable level of missingness (i.e., CRP) as shown in the supplementary file (*eFigure3)*. This demonstrates the validity of assuming that selected clinical variables (although not necessarily all variables) can be treated as normal.

In addition to the above variables which were retained in the final model, many other variables were also considered, but were ultimately dropped from the model. In refining our model, each variable was evaluated based on their distribution and relationship with the outcomes, both bivariate and bivariate analyses, as well as considering clinical relevance, such as normal or critical values for vital signs and lab results. Categories were adjusted to optimize these relationships, often revised in the context of their clinical relevance to ensure maximum relevance and minimal complexity. Consequently, variables were excluded for reasons such as small effect sizes, substantial missing data, or limited patient impact. The complete variable list, and a description of the methods used for construction of the model, appears in the supplemental file in in *etable2*, and *eMethods2.*

### Statistical analyses

We began by examining the univariate distribution of all variables. We then examined each of the predictor variables in a binary fashion, with the exception of the Elixhauser variables, since it was our intention to retain the entire Elixhauser Index as the basis for our model. We used the binary analyses in part to optimize the categorization of each variable. For example, when examining pulse (*beats per minute*), we began with five categories (< 80, 80–99, 100–119, 120–149, 150+), but later combined several categories based on them having similar results in bivariate analyses. Our final model contained only three levels for pulse: <100, 100–119, and 120+. To produce the extended model and to further validate it, we randomly divided our dataset 70/30 into derivation and validation sets. We used the derivation set to derive our model coefficients for each of the outcomes, and then forced those coefficients onto the validation set to check that model fit did not decrease (which would have indicated overfitting).

For the continuous outcome (log LOS), fit was characterized using R^2^. For the binary outcomes, model fit was characterized using the c-statistic. Net Reclassification Index (NRI) and Integrated Discrimination Improvement (IDI) were used to further examine model performance in comparison to the base Elixhauser model. The NRI includes cross-tabulation of categories of predicted risk for two prediction models to assess changes in risk classification, and quantify the improvement with the addition of the new predictors compared to the baseline model [24, 25]. The IDI, as a complementary measure, reflects the change in discrimination slopes between the two models. While the NRI emphasizes the reclassification of individuals, the IDI offers a more focused assessment of the overall improvement in model discrimination, especially when changes in risk prediction are not fully captured by categorical shifts alone [24, 26]. Additionally, with the binary outcomes, we used a Hosmer-Lemeshow test to examine model calibration, or whether the model systematically overestimates risk in lower-risk or in higher-risk groups [27].

## Results

A total of 55,945 hospitalizations were documented, with some patients hospitalized more than once. 13% of hospitalizations involved increased care, 6.9% ended in an in-hospital death, and 15% of patients were readmitted to SZMC within 30 days of discharge. The median LOS was 4 days (IQR: 2,7).

Table 1 shows that we achieved balance by randomly selecting the derivation and validation sets. The derivation set contained 39,161 hospitalizations, and the validation set 16,784 hospitalizations. None of the between-group differences are statistically significant at the 0.05 level, other than elective vs. urgent admissions.

**Table 1:** Patient characteristics

Sample				
Characteristic	Overall *N* = 55,945^a^	Validation *N* = 16,784^a^	Derivation *N* = 39,161^a^	*p*-value^b^
**Sex** (n; %)				> 0.9
Male	26,030 (47%)	7,810 (47%)	18,220 (47%)	
Female	29,915 (53%)	8,974 (53%)	20,941 (53%)	
**Age**				> 0.9
18–69	22,160 (40%)	6,664 (40%)	15,496 (40%)	
70–79	12,264 (22%)	3,687 (22%)	8,577 (22%)	
>80	21,521 (38%)	6,433 (38%)	15,088 (39%)	
**Number of Elixhauser Comorbidities** (n; %)				0.5
0	13,455 (24%)	3,970 (24%)	9,485 (24%)	
1	11,149 (20%)	3,337 (20%)	7,812 (20%)	
2–4	25,155 (45%)	7,598 (45%)	17,557 (45%)	
5+	6,186 (11%)	1,879 (11%)	4,307 (11%)	
**In-Hospital Mortality** (n; %)	3,865 (6.9%)	1,173 (7.0%)	2,692 (6.9%)	0.6
**Readmission in 30 days** (n; %)	8,620 (15%)	2,574 (15%)	6,046 (15%)	0.8
**Increased Care** (n; %)	7,489 (13%)	2,191 (13%)	5,298 (14%)	0.13
**Hospital Length of Stay** (Days) (Median; IQR range)	4.1 (5.7)	4.1 (5.5)	4.1 (5.7)	0.2

Characteristics for 55,945 internal medicine admissions to Shaare Zedek Medical Center, Israel (2016–2019). Hospitalizations are divided into derivation and validation sets. Cell count (%) are presented

^a^ Median (IQR) or Frequency (%)

^b^ Chi-square test; One-way ANOVA

A majority of hospitalizations were males (53%), with an average age of 70.9 years (SD 17.8). 76% of hospitalizations were assigned at least one Elixhauser diagnosis group, 56% had at least two diagnosis groups, and 11% had five or more. Some variables were almost never missing, such as age. Others were missing more often, such as respiratory rate and serum c-reactive protein, which were missing for 79% and 44% of hospitalizations, respectively.

Table 2 compares the frequency of patient-level characteristics between the derivation and validation sets. The table shows that the groups were balanced, and also describes the patient population overall. e*Table3* in the supplemental file shows the results of the multivariate regressions for each of the outcomes, with adjusted odds ratios (for logistic regressions) and adjusted beta coefficients (for the linear regression). For example, a patient with a systolic BP of less than 80 mm/Hg had adjusted odds of 2.14 to die during hospitalization, compared with patients with systolic BP 80 mm/Hg or higher. The linear regression modeled the outcome of log LOS; therefore, beta coefficients cannot simply be interpreted as being in units of days.

**Table 2:** Patient-level predictors

Sample				
Characteristic	Overall *N* = 55,945^a^	Validation *N* = 16,784^a^	Derivation *N* = 39,161^a^	*p*-value^b^
**Heart rate** *(Beats per minute)*				0.3
< 100 [Ref]	42,689 (76%)	12,750 (76%)	29,939 (76%)	
100–119	9,412 (17%)	2,845 (17%)	6,567 (17%)	
> 120	3,844 (6.9%)	1,189 (7.1%)	2,655 (6.8%)	
**Systolic Blood Pressure** *(mmHg)*				0.9
< 80	945 (1.7%)	281 (1.7%)	664 (1.7%)	
> 80 [Ref]	55,000 (98%)	16,503 (98%)	38,497 (98%)	
**Temperature** *(Celsius)*				0.7
< 35	108 (0.2%)	29 (0.2%)	79 (0.2%)	
35-39.9 [Ref]	55,763 (100%)	16,734 (100%)	39,029 (100%)	
> 40	74 (0.1%)	21 (0.1%)	53 (0.1%)	
**Blood Oxygen Saturation** *(%)*				0.2
< 80	1,053 (1.9%)	335 (2.0%)	718 (1.8%)	
> 80 [Ref]	54,892 (98%)	16,449 (98%)	38,443 (98%)	
**1** ^**st**^ **Creatinine** *(mg/dL)*				0.6
< 1.2 [Ref]	38,509 (69%)	11,526 (69%)	26,983 (69%)	
1.2–1.79	9,280 (17%)	2,823 (17%)	6,457 (16%)	
> 1.8	8,156 (15%)	2,435 (15%)	5,721 (15%)	
**1** ^**st**^ **Albumin** *(g/dL)*				> 0.9
< 3	9,578 (17%)	2,865 (17%)	6,713 (17%)	
3-3.49	11,422 (20%)	3,433 (20%)	7,989 (20%)	
> 3.5 [Ref]	34,945 (62%)	10,486 (62%)	24,460 (62%)	
**1** ^**st**^ **Hematocrit** *(%)*				0.2
< 30	8,240 (15%)	2,401 (14%)	5,839 (15%)	
30-49.9 [Ref]	46,934 (84%)	14,155 (84%)	32,779 (84%)	
> 50	771 (1.4%)	228 (1.4%)	543 (1.4%)	
**1** ^**st**^ **CRP** *(mg/dL)*				0.9
< 5 [Ref]	41,080 (73%)	12,285 (73%)	28,795 (74%)	
5-19.9	11,394 (20%)	3,451 (21%)	7,943 (20%)	
20–30	2,669 (4.8%)	805 (4.8%)	1,864 (4.8%)	
> 30	802 (1.4%)	243 (1.4%)	559 (1.4%)	
**1** ^**st**^ **Sodium** *(mEq/L)*				0.6
< 130	3,859 (6.9%)	1,183 (7.0%)	2,676 (6.8%)	
130-142.9 [Ref]	48,298 (86%)	14,463 (86%)	33,835 (86%)	
> 143	3,788 (6.8%)	1,138 (6.8%)	2,650 (6.8%)	
**1** ^**st**^ **Potassium** *(mEq/L)*				0.6
< 5 [Ref]	50,560 (90%)	15,152 (90%)	35,408 (90%)	
> 5	5,385 (9.6%)	1,632 (9.7%)	3,753 (9.6%)	
**1** ^**st**^ **Glucose** *(mg/dL)*				0.5
< 70	711 (1.3%)	225 (1.3%)	486 (1.2%)	
70-129.9 [Ref]	33,753 (60%)	10,138 (60%)	23,615 (60%)	
130-199.9	13,620 (24%)	4,037 (24%)	9,583 (24%)	
> 200	7,861 (14%)	2,384 (14%)	5,477 (14%)	
**Elective Admission**				< 0.001
0 [Ref]	48,091 (86%)	14,555 (87%)	33,536 (86%)	
1	7,854 (14%)	2,229 (13%)	5,625 (14%)	
**Transport Vehicle**				0.7
Other [Ref]	29,081 (52%)	8,682 (52%)	20,399 (52%)	
Ambulance	26,368 (47%)	7,953 (47%)	18,415 (47%)	
ICU Ambulance	496 (0.9%)	149 (0.9%)	347 (0.9%)	
**Time Past Last Hospitalization**				0.12
< 30	9,152 (16%)	2,694 (16%)	6,458 (16%)	
30–89	5,228 (9.3%)	1,624 (9.7%)	3,604 (9.2%)	
90 or Never [Ref]	41,565 (74%)	12,466 (74%)	29,099 (74%)	
**Num of Prior Hospitalizations**				0.7
< 3 [Ref]	33,959 (61%)	10,206 (61%)	23,753 (61%)	
+ 3	21,986 (39%)	6,578 (39%)	15,408 (39%)	

Characteristics for 55,945 internal medicine admissions to Shaare Zedek Medical Center, Israel (2016–2019). Hospitalizations are divided into derivation and validation sets. Cell count (%) are presented

^a^ Frequency - n (%)

^b^ Pearson’s Chi-squared tests

The variables included in our final model were moderately or highly predictive of the study outcomes (all findings discussed here were significant at the *p* < 0.05 level). The strongest predictors of in-hospital mortality were a Norton mental scale of 1 (AOR:5.40, CI:4.05–7.21) and CRP > 30 mg/dL (AOR:3.39, CI:2.65–4.33). Other than HIV, which was uncommon (16 patients), the leading predictors for increased care were blood oxygen saturation < 80% (AOR:2.83, CI: 2.39–3.35), CRP > 30 mg/dL (AOR:2.46, CI: 2.00-3.01) and temperature < 35 Celsius (AOR:2.44, CI:1.42–4.14). The strongest predictors of 30-day readmission were less than 30 days since the last hospitalization (AOR:1.62, CI: 1.50–1.75) and Albumin 3–3.49 g/dL, (AOR: 1.38, CI: 1.28–1.49). The strongest predictors of log-LOS were Albumin < 3 g/dL and CRP > 30 mg/dL (adjusted beta-coefficients:0.56, CI:0.55–0.58 and 0.28, CI:0.23–0.33 respectively).

When predicting outcomes in the validation group based on beta coefficients obtained from derivation group, only minor changes were observed between the derivation and validation sets, suggesting that there was no overfitting of the models to the derivation group. *eFigure*4 compares the receiver-operator curves from the derivation and validation sets.

**Fig. 1:**
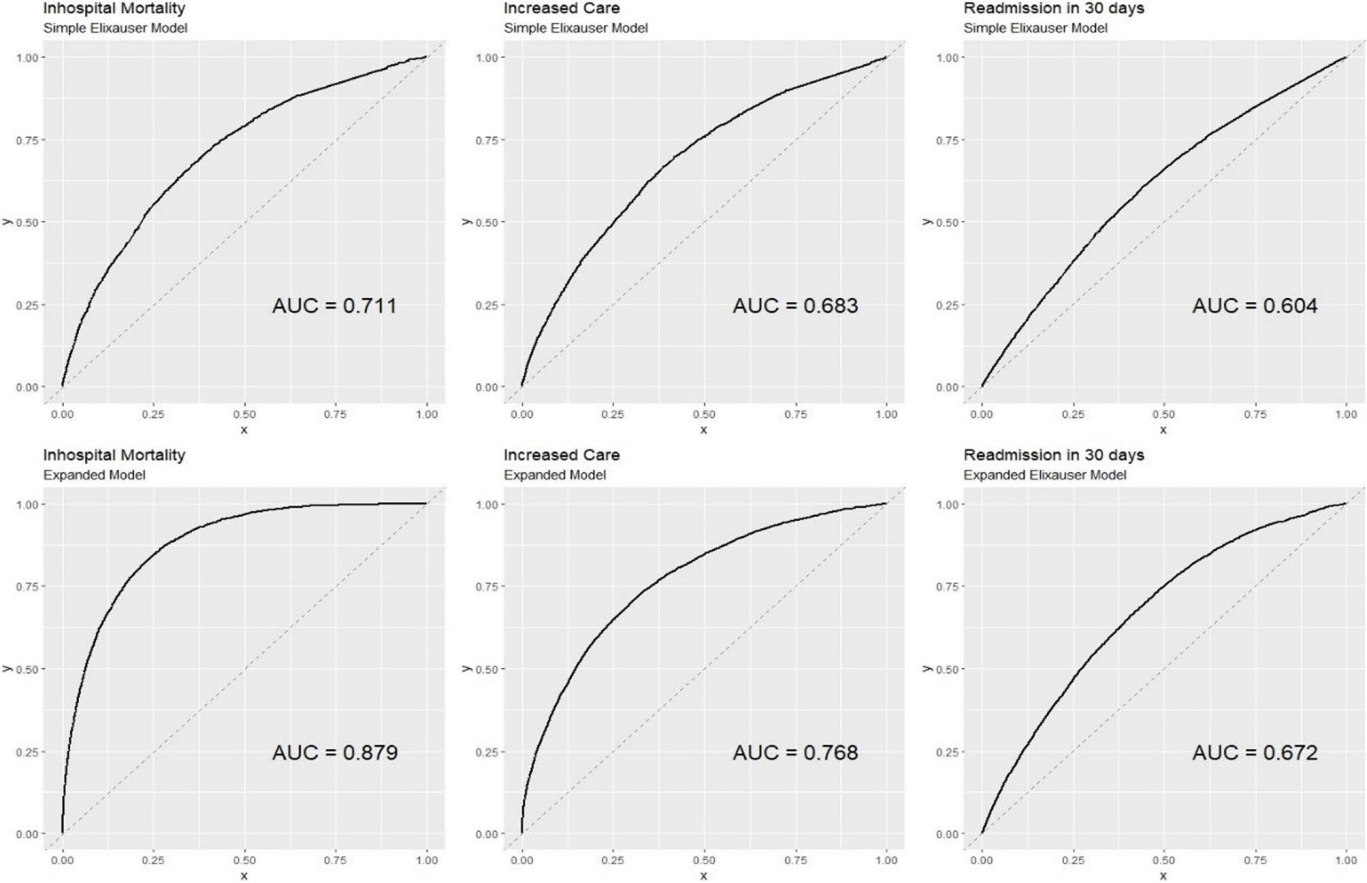
Comparison of AUC and c-statistic between enhanced model and the base Elixhauser model

Table 3 shows the performance of the expanded model compared to the basic Elixhauser model. Among the binary outcomes, the most predictive model was the one to predict in-hospital mortality (c-statistic 0.879) followed by increased care and 30-day Readmission (c-statistics 0.768 and 0.672 respectively). This is further demonstrated in Fig. 1. The expanded model performed meaningfully better than the base Elixhauser model for predicting all outcomes, with the difference being as large as 16.8% better in absolute terms (for predicting mortality) and as little as 6.8% better (for predicting 30-day readmission).

**Table 3:** Tests of model fit and calibration

	In Hospital Mortality	Readmission in 30 days	Increased Care	LoS (log)
**c-statistic**				
* Simple model*	0.711	0.604	0.683	-
* Expanded model*	0.879	0.672	0.768	-
**Adjusted R** ^**b**^				
* Simple model*	-	-	-	0.101
* Expanded model*	-	-	-	0.281
***NRI*** ^***a***^	0.431	0.142	0.351	-
***NRI***_***event***_^***b***^	0.421	0.075	0.212	-
***NRI***_***non−event***_^***b***^	0.010	0.067	0.139	-
***IDI*** ^***c***^	0.167 [0.155–0.180]	0.029 [0.026–0.032]	0.094 [0.086–0.102]	

Calibration of the models for in-hospital mortality; Readmission in 30 days; Increased care; and LOS as perceived by c-statistics NRI and IDI for logistic regressions and adjusted R^2^ for linear regressions

^a^ sum of NRI _e_ and NRI _ne_

^b ^% of patients assigned a more accurate risk score

The percent variance explained for length of stay (LOS), as indicated by R^2^ for linear regression, increased substantially from 0.101 with the base model to 0.281 with the enhanced model.

We also used the Net Reclassification Index (NRI) to better understand the extent to which the added information improved the prediction of the logistic models. The NRI_e_ of 0.42 signifies that 42% of patients who experienced in-hospital mortality were correctly reclassified into a higher-risk group when compared to their classification based on the simple model. Moreover, the extended model yielded an NRI_e_ of 0.21 and 0.07 for increased care and readmission prediction, respectively, indicating improved classification accuracy for these outcomes as well. Additionally, the extended model also effectively assigned patients without an event to a lower-risk group, as evidenced by an NRI_ne_ of 0.01 0.06 and 0.13 for in-hospital mortality, increased care and readmission respectively.

Similarly, when evaluating the extended model using the IDI, a slight improvement was observed, with IDI values of 0.16, 0.09, and 0.02 for the models predicting in-hospital mortality, increased care, and readmission, respectively. These relatively low values suggest that while there is some improvement in model performance, the overall enhancement in discrimination remains modest.

The Hosmer-Lemeshow test showed good overall model calibration for the three logistic models. The mortality model did show a tendency to overestimate the risk of death in the first and second deciles. Detailed results of the Hosmer-Lemeshow test can be found in *eTable1*.

## Discussion

In this study, we validated a version of the Elixhauser model, by adding information such as vital signs and lab tests available at the time of admission or arrival to the medical ward. Using a dataset of approximately 56,000 admissions to internal medicine from a large, tertiary referral hospital, we showed that this enhanced model was considerably more predictive than the original Elixhauser model. In particular, the c-statistic to predict in-hospital mortality (0.879) is a level of prediction not often achieved.

The most challenging outcome to predict among the four we studied was 30-day readmission, which had the lowest c-statistic. One potential explanation for this finding is that we only captured rehospitalizations to SZMC. However, it is worth noting that most patients discharged from SZMC are also readmitted to SZMC [18], minimizing the magnitude of this issue. In a systematic review encompassing 41 articles on predictive models for 30-day readmission using electronic medical record data, a broad range of c-statistic values were reported, spanning from 0.52 to 0.90. Machine learning models exhibited an average c-statistic of 0.74, while traditional regression models had an average of 0.71 [28]. Our c-statistic of 0.672 was somewhat lower than this average, but our model still achieved a reasonable level of discrimination in predicting 30-day readmissions.

Our model has several potential applications for future use. The primary goal of the study was to develop a tool for adjusting patient risks, enabling health systems to compensate healthcare providers based on the patient’s risk level for specific outcomes. This calculated risk reflects and accounts in certain ways for the costs of the patient’s hospitalizaion. Additionally, the model can be used to classify patients and assist in clinical decision-making based on their risk for various hospitalization outcomes. For example, it may help identify patients who require admission to the ICU. Furthermore, it can stratify patients according to their risk levels, facilitating comparisons between different healthcare centers for health policy, decision-making, and research purposes. The variables that populated our model are available at the time of hospitalization, allowing them to be used for allocation decisions, namely decisions about whom to admit and to which setting. In addition, because this model relies on information available at the time of initial presentation, it is clear that the patient’s severity, as estimated using this score, does not reflect poorly on care that they received during this hospitalization. Therefore, it could also be used for risk-adjustment and differential payments by complexity.

Another advantage of the model that we describe here is that the variables that we used are widely available in many settings. Vital signs, basic laboratory tests, and comorbid condition diagnoses are available as structured data in most, if not all hospital settings, at least in developed countries. Therefore, our score could be used in a wide variety of settings. It would be worthwhile to directly establish that it works as well in other datasets as it worked in this one.

There have been previous efforts to improve prediction of hospitalization-related outcomes. Most previous studies which developed an expanded risk-adjustment hospitalization outcome models did so for a single hospitalization outcome (i.e., in-hospital mortality), or among patients who all have a specific medical condition [29–34]. For example, Pine et al. found that in five health conditions and three surgical ones, adding laboratory data to comorbid conditions improved the performance of risk-adjustment models. Additional clinical data elements, in that study, added substantially less to model prediction [29]. Amarasingham et al. developed a risk-adjustment model using data present on admission to predict mortality and readmission in 30 days for heart failure patients and achieved a c-statistic of 0.86 and 0.72 respectively, both of which outperformed prior models [34]. The level of performance of our model echoes what was achieved by Amarasingham. However, the Amarasingham model is only for patients admitted with heart failure, while our model was developed and validated with a diverse population of patients admitted to internal medicine.

Our study has important strengths, especially when compared with other studies that have had similar goals. Previous studies have often focused on specific conditions or used limited data inputs, and therefore may not generalize well across different settings, which may limit their real-world usefulness. Our study contributes to this field of research by addressing these limitations, specifically including multiple inpatient hospitalization outcomes without being limited to a specific diagnosis. The use of vital signs, basic laboratory tests, and comorbid condition diagnoses, which are commonly available in most hospital settings, further enhances the model’s practicality and adaptability. Importantly, our model’s reliance on initial presentation data ensures an unbiased assessment of patient severity, making it suitable for risk-adjustment and differential payments by complexity.

Our study also has limitations. The main limitation of our study is that it is a single center study, which should further be validated across multiple centers to validate the model’s effectiveness and accuracy in diverse patient populations. Another limitation of our model is the use of automated data provided by the medical institution, in terms of accuracy and completeness of the data. This is particularly relevant to the documentation of medical diagnoses using ICD-9 codes, which can often be deficient. This limitation would be shared by any risk-adjustment model that relies on automated data. To help mitigate this, extensive work was undertaken to enhance the data’s accuracy.

A concern about this is that if the model is used as a basis for differential payment by complexity, this may create an incentive for the hospital to manipulate and up-code the data to try to receive higher payments [35–38]. This limitation can be addressed, in part, by selecting variables that are harder to “game”. In particular, the Norton score, because it is somewhat subjective, could be subject to gaming. We therefore also examined a version of the model without this score (*eTable4*), which was almost as predictive as the full model. We think it is less likely that vital signs and lab results would be subject to gaming. This is also the reason that we assumed that missing lab values are normal – because this assumption does not create perverse incentives to manipulate the data.

Despite these limitations, our study demonstrated that the model we developed is meaningfully better than the basic Elixhauser model, through the addition of additional clinical information. Future directions for research include demonstrating the applicability of this model at the national level (in Israel) and also validating it in settings outside Israel. It is also worthwhile to consider using this model as the basis for improving admission decisions or for differential payment by complexity – these too would be fitting topics for future research.

## Conclusions

We have shown that it is possible to develop a model that includes demographic, clinical and diagnostic information that will predict hospitalization outcomes well. This model has a variety of uses and, among other things, it can be used as a basis for appropriate compensation of hospitals based on patient complexity.

## Supplementary Information

Supplementary Material 1.

## Data Availability

We will share our statistical code and study protocol upon request. SZMC data are available upon reasonable request and after completing a data use agreement.

## Abbreviations

CCI: Charlson Comorbidity Index
ECM: Elixhauser Comorbidity Measure
ICU: Intensive Care Unit
BiPAP: Bi-level Positive Airway Pressure
SZMC: Shaare Zedek Medical Center
LOS: Length of stay
