# Supplementary material for: Incorporating clinical and demographic data into the Elixhauser Comorbidity Model: deriving and validating an enhanced model in a tertiary hospital’s internal medicine department
Source: BMC Health Serv Res. 2024 Dec 5;24:1523. doi: 10.1186/s12913-024-11663-z (PMC11619165; doi:10.1186/s12913-024-11663-z)
Supplement: Supplementary file 1 — Supplementary Material 1. [file 12913_2024_11663_MOESM1_ESM.docx]

**Supplements**

1. **eMethods1:** List of Vasopressors
2. **eMethods2:** Detailed Methodology for Model Creation
3. **eFigure1:** Sensitivity Analysis of Patient Based Obseravation Impact on Model Performance
4. **eFigure2:** Sensitivity Analysis of Length of Stay Exclusions and Impact on Model Performance
5. **eFigure3:** Sensitivity Analysis Missing as Normal vs Imputed Models
6. **eFigure4:** Comparison of Derivation and Validation sets.
7. **eTable1:** Complete List of Variables Considered for Inclusion in the Model
8. **eTable2:** Full Results of Hosmer-Lemeshow tests
9. **eTable3:** Multivariable Regression Expanded Model
10. **eTable4:** Multivariable Regression Expanded Model (without Norton Scale)

**eMethods 1: List of Vasopressors**

The following vasopressor medications were used in our study, and constituted our list of vasopressors:

- Adrenaline
- Dobutamine
- Dopamine
- Milrinone
- Noradrenaline
- Phenylephrine
- Vasopressin

Vasopressors must be given by intravenous infusion, and not intramuscularly, orally, or by another route. Can be given at any time in the hospitalization, including in the emergency department, medical ward, intensive care unit, or intermediate care unit. Other vasopressors exist, but were not observed in our dataset (e.g., Angiotensin, Terlipressin).

**eMethods2:** Detailed Methodology for Model Creation

This document outlines the structured approach we used for developing a predictive risk adjustment model for patient outcomes within internal medicine wards. It serves as a step-by-step guide, detailing each phase of the work from conceptualization to model validation. This guide is intended for researchers and healthcare professionals aiming to replicate or adapt our study in various healthcare settings.

**Stage 1: Conceptualization**

- Identifying the need for a model
  - **Objective assessment**: Identify key patient outcomes suitable for predictive modeling, such as a risk adjustment model to predict patient outcomes.
  - **Outcome Selection** : Choose outcomes relevant to patient care and hospital management, such as length of stay, in-hospital mortality, readmission rates, and need for increased care/escalation of care.
- Defining the target population on which the model will be applied.
  - **Population Scope:** Define the patient population (e.g., patients admitted to internal medicine) to ensure the model's applicability and relevance, for the desired population.

**Stage 2: Data Preparation**

- Selecting variables
  - **Initial Selection**: Choose variables based on availability, reliability, and their potential for internal and external validation. Focus on data that can be easily and accurately collected. Whenever possible, focus on variables that are complex or difficult to game by the various stakeholders, and that can be validated from another source.
  - **Clinical and Operational Relevance** : Prioritize variables that are clinically significant and operationally feasible for the desired model. In our case we focused on variables that can be collected in the emergency department at the time of admission, to help predict admission outcomes.
- Managing missing data
  - **Assessment of missing data:** Evaluate the extent of missing data for each variable.
  - **Strategy for missing data:** Decide on a strategy (e.g., treating missing values as normal, using average/median values, or applying multiple imputation) based on the model's purpose and the nature of the missing data.

**Stage 3: Variable Categorization and Modification**

- Categorization of continuous variables
  - **Initial Categorization:** Divide continuous variables into categories based on clinical judgment and the variable's distribution, aiming to create meaningful groups.
  - **Univariate Model Examination:** Assess the variables' behavior in univariate models and adjust categorizations as necessary to enhance predictive significance and model performance.
- Finalizing variables for the model
  - **Variable Evaluation:** Reassess and finalize the selection of variables for inclusion in the model based on their performance in preliminary analyses.
  - **Omit Variables:** Exclude variables that exhibit small effect sizes, high levels of missing data, or those that affect only a few patients.

**Stage 4: Model Construction**

- Division of data
  - **Create Split Sample:** Divide the dataset into training and validation sets to facilitate model development and subsequent internal validation. Ensures a large enough training sample to capture the complex relationships inherent in modeling patient outcomes, thus minimizing the risk of underfitting. The remaining data will be the validation set, enabling internal validation of the model's coefficients.
- Regression model development
  - **Model Building:** Construct separate regression models for each outcome of interest using the training set.
  - **Validation and Adjustment:** Apply the derived coefficients to the validation set to test the model's predictive accuracy and make necessary adjustments if overfitting is detected.

**Stage 5: Validation and Refinement**

- Model testing
  - **Accuracy Assessment:** Evaluate the model's accuracy in predicting outcomes using the validation set.
  - **Performance Metrics and Error Analysis:** Apply the model to the validation set and use appropriate metrics (e.g., RMSE for continuous outcomes, AUC-ROC for categorical outcomes) to evaluate prediction accuracy. Compare performance against baseline models to contextualize improvements.
  - **Refinement:** Based on validation outcomes, refine the model by adjusting variables, algorithms, or parameters to improve predictive accuracy and minimize biases. Conduct error analysis to identify and correct mispredictions.
  - **Statistical Significant and Calibration:** Calculate confidence intervals for accuracy metrics to assess statistical significance and use calibration to evaluate how well the model's predicted probabilities match actual outcomes, ensuring reliable predictions.

**eFigure1:** *Comparison of receiver operator curves and c-statistics between the hospitalization based observation and the sensitivity analysis model were the patient last visit was defined as*
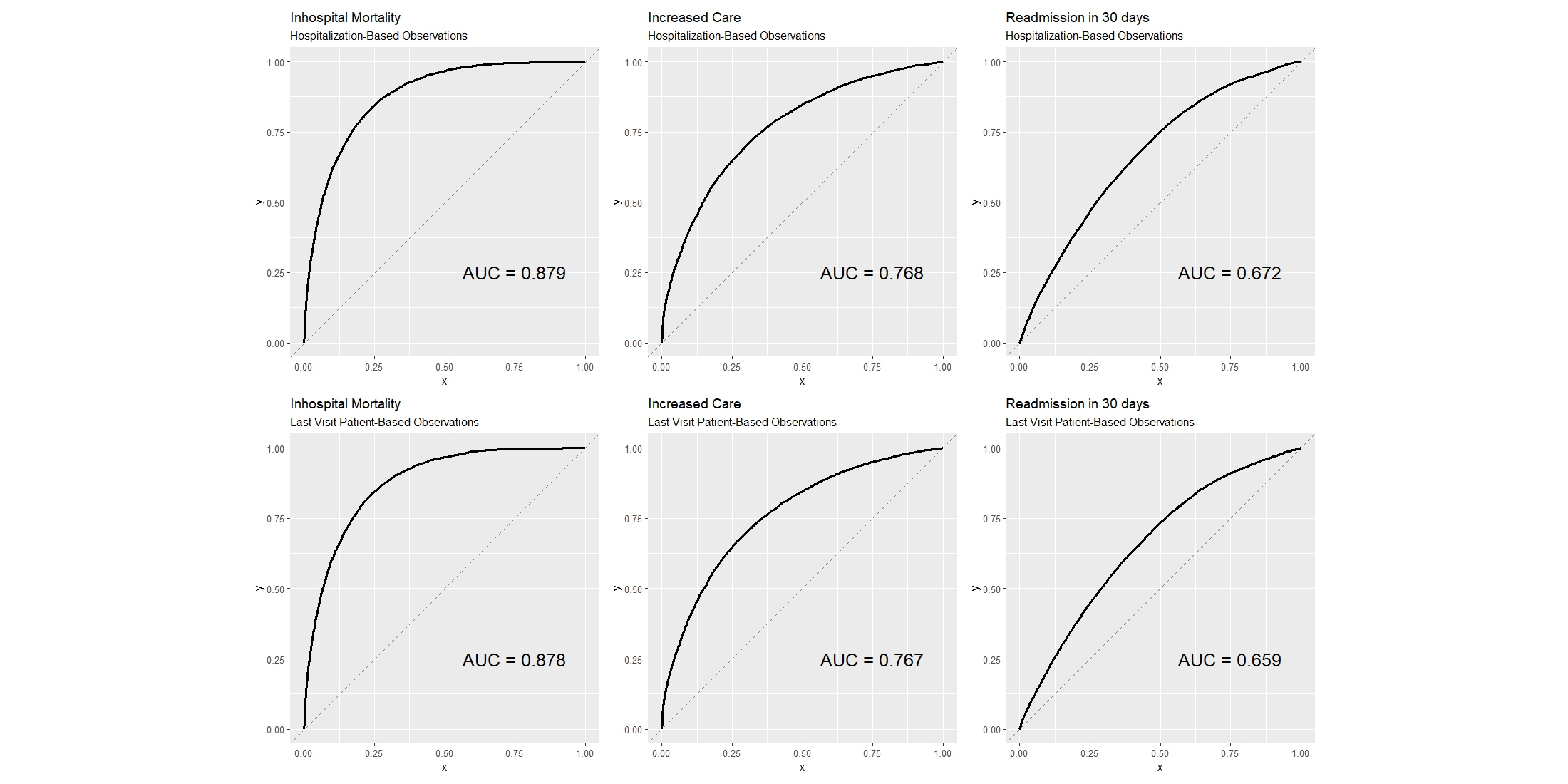
*the unit of obseravation. The analysis shows minimal changes in model performance.*


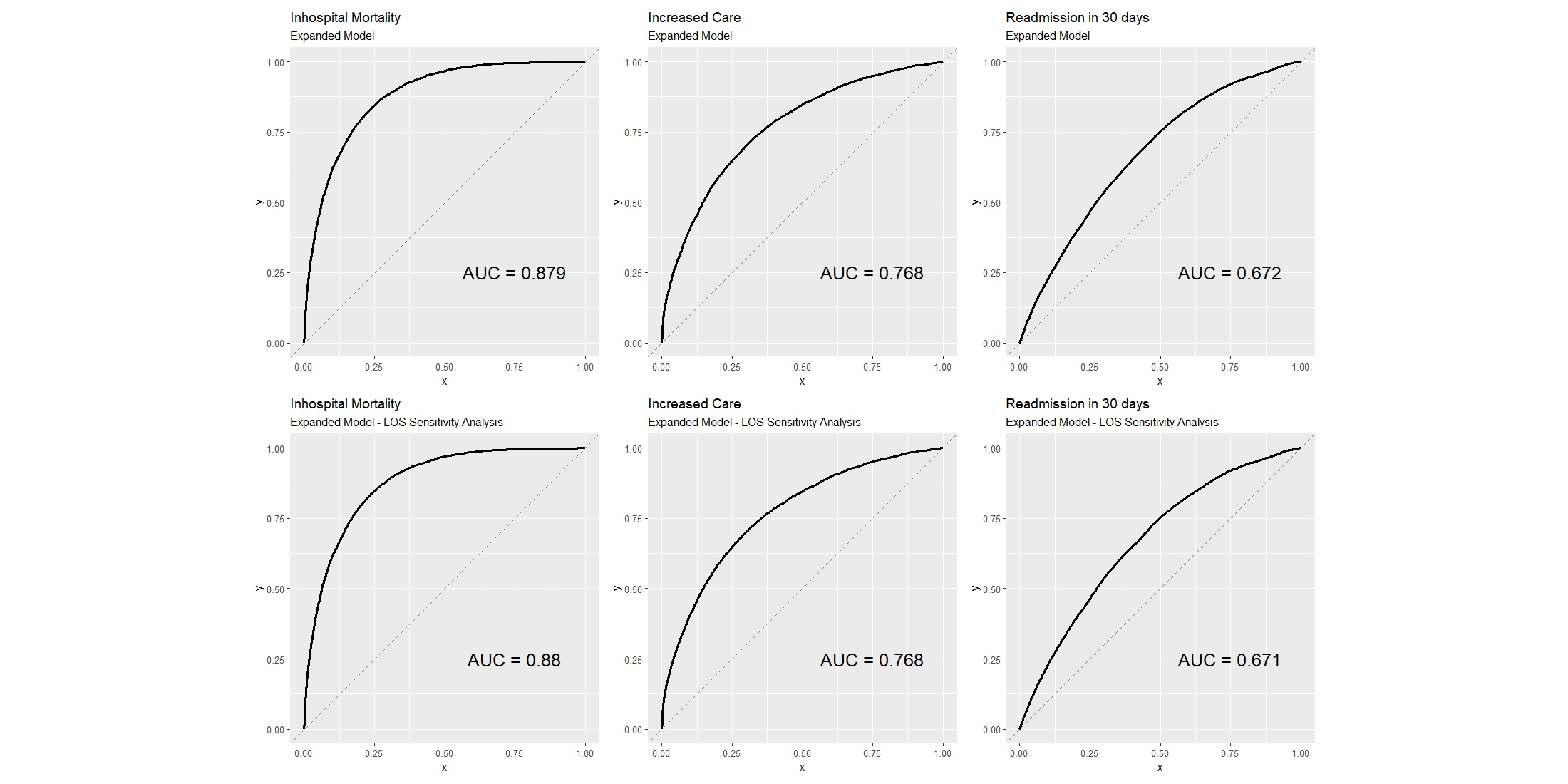
**eFigure2:**  *Comparison of receiver operator curves and c-statistics between the expanded model and the sensitivity analysis model, where hospital stays longer than 150 days were excluded. The analysis shows minimal changes in model performance.*


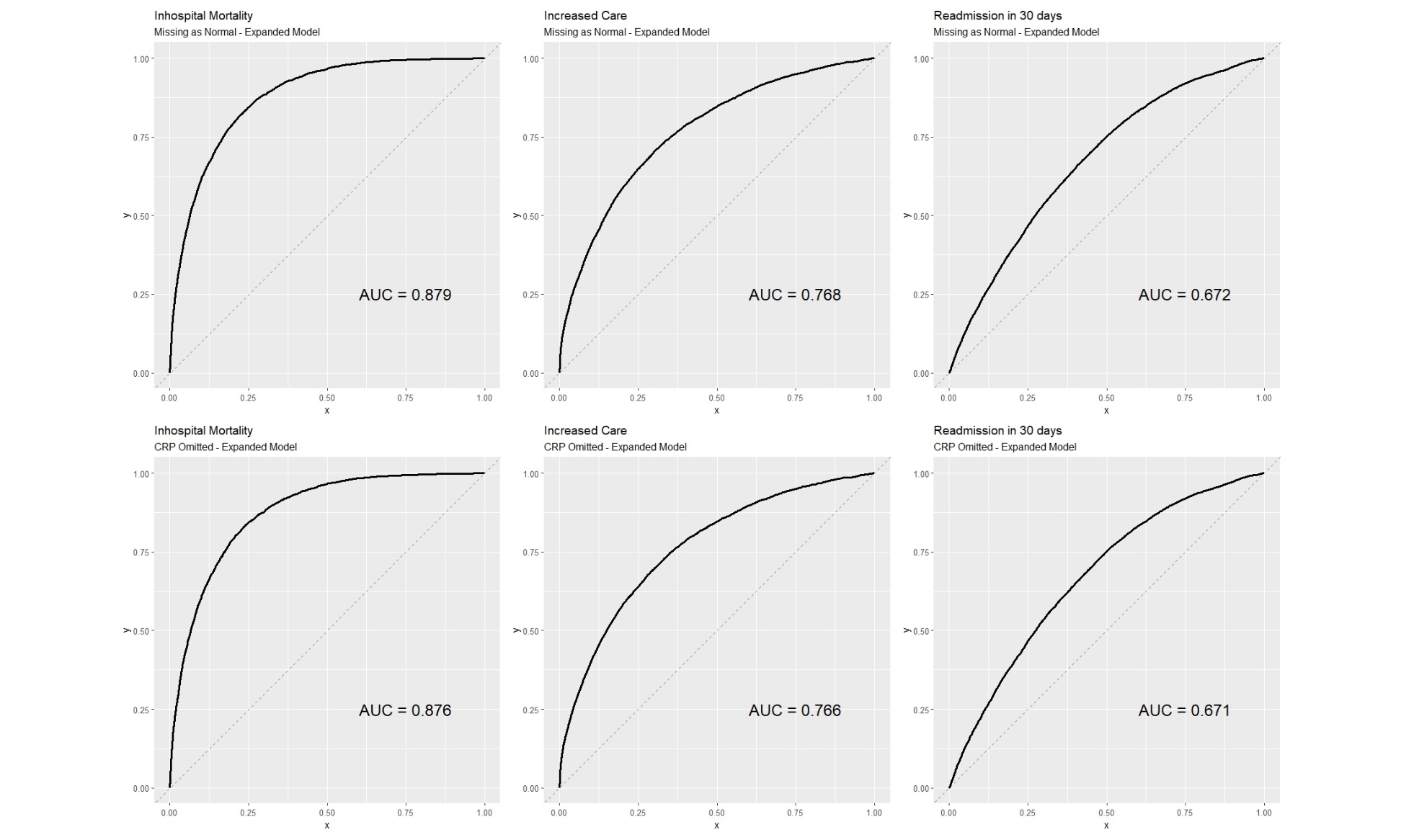
**eFigure3:**  *Comparison of receiver operator curves and c-statistics obtained from the Missing as normal model, compared with the omitted model were CRP variable (which had a considerable level of missingness) was removed from the model.*

**eFigure4:**  *Comparison of receiver operator curves and c-statistics obtained from the derivation and validation sets.*


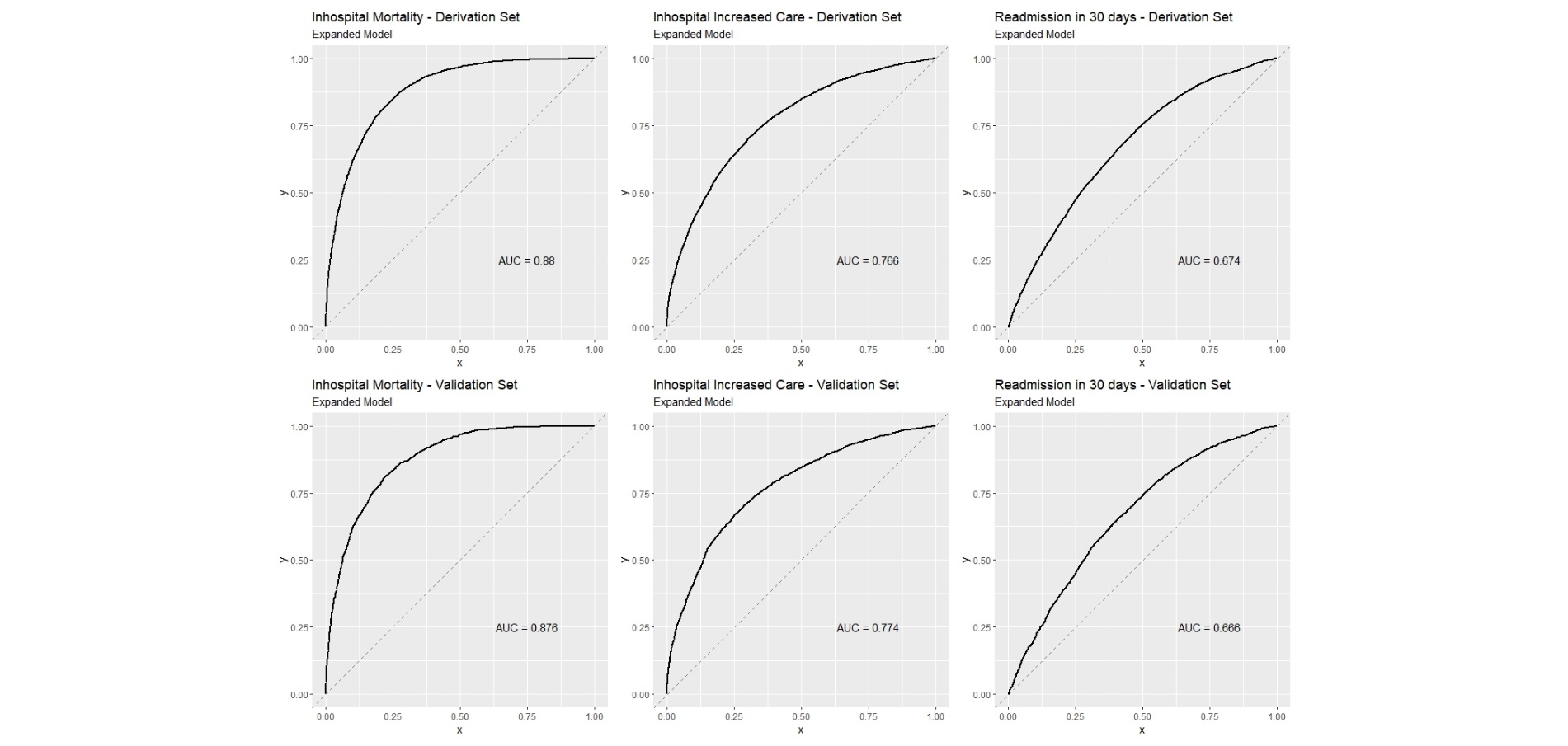


**eTable1: Complete List of Variables Considered for Inclusion in the Model:**

| Variable | **Missing** | **Median** | **Mean** | **Min** | **Max** |
| --- | --- | --- | --- | --- | --- |
| **Age** | - | 74 | 70.93 | 18 | 118 |
| **Sex** | - | - | - | - | - |
| **Body Mass Index** | 19,769 (35.3%) | 26.18 | 27.05 | 10.02 | 87.00 |
| **1^st^ Pulse** | 1,175 (2.1%) | 83.00 | 85.83 | 0 | 247 |
| **1^st^ Systolic Blood Pressure** | 1,203 (2.15%) | 130.0 | 131.8 | 0 | 262 |
| **1^st^ Diastolic Blood Pressure** | 1,238 (2.21%) | 74 | 74.03 | 0 | 174 |
| 1. 1^st^ Calculated Mean Arterial Blood Pressure | 1,244 (2.22%) | 93.0 | 93.28 | 0 | 196.3 |
| 1. 1^st^ Temp | 13,201 (23.6%) | 36.7 | 36.88 | 25.9 | 41.5 |
| 1. 1^st^ Saturation | 5,258 (9.39%) | 95.0 | 93.82 | 0.0 | 100.0 |
| 1. 1^st^ Respiratory Rate | 44,400 (79.36%) | 16.0 | 17.55 | 1.0 | 60.0 |
| 1. Do Not Resuscitate Order | 52,669 (94.14%) | - | - | - | - |
| 1. Do Not Intubate Order | 52,773 (94.33%) | - | - | - | - |
| 1. Type of admission (elective/urgent) | 0 | - | - | - | - |
| 1. Norton scale (Physical) | 3,128 (5.59%) | - | - | - | - |
| 1. Norton scale (Mental) | 3,128 (5.59%) | - | - | - | - |
| 1. Norton scale (Mobility) | 3,128 (5.59%) | - | - | - | - |
| 1. Norton scale (Activity) | 3,128 (5.59%) | - | - | - | - |
| 1. Norton scale (Incontinent) | 3,128 (5.59%) | - | - | - | - |
| 1. Norton scale (Total score) | 3,128 (5.59%) | - | - | - | - |
| 1. Fall risk scale | 32,500 (58.09%) | - | - | - | - |
| 1. Point of Origin (home, nursing home, etc..) | 14,619 (26.13%) | - | - | - | - |
| 1. Mother tongue |  | - | - | - | - |
| 1. Transport vehicle (ambulance, car, etc.) | 7,856 (14.04%) | - | - | - | - |
| 1. Serum Creatinine | 4,083 (7.29%) | 0.97 | 1.345 | 0.11 | 35.59 |
| 1. Serum Albumin | 20,087 (35.9%) | 3.3 | 3.29 | 0.7 | 5.6 |
| 1. Serum Hematocrit | 4,459 (7.97%) | 37.1 | 36.6 | 2.7 | 76.4 |
| 1. Serum c-reactive protein | 24,862 (44.44%) | 4.47 | 7.83 | 0.02 | 64.16 |
| 1. Serum Sodium | 4,139 (7.39%) | 137 | 136.9 | 100.0 | 170.0 |
| 1. Serum Potassium | 5,946 (10.62%) | 4.1 | 4.19 | 1.1 | 9.5 |
| 1. Serum Glucose | 4,112 (7.35%) | 120.0 | 146.8 | 8.0 | 1445.0 |
| 1. Ulcer noted within 24 hours of hospitalization | - | - | - | - | - |
| 1. Number of prior hospitalizations | 28,296 (50.57%) | 8.0 | 11.34 | 1.0 | 50.0 |
| 1. Number of hospitalizations in the last year | 28,296 (50.57%) | 1.0 | 1.90 | 0 | 35.0 |
| 1. Time since last hospitalization | 30,126 (53.84%) | 64.5 | 203.1 | 0.0 | 1460.0 |
| 1. Length of stay of prior hospitalization | 28,296 (50.57%) | 4.01 | 6.48 | 0.34 | 35.08 |
| 1. Mean length of stay of all prior hospitalizations | 28,296 (50.57%) | 90.6 | 117.6 | 2.8 | 8736.8 |
| 1. Rate of hospitalizations per year during study period | 28,296 (50.57%) | 62.9 |  |  |  |

**eTable2: Multivariable Regression**

*eTable2 – Multivariable regression results for In-hospital Mortality, Escalation of Care, Readmission in 30 days, and LoS. Derived from derivation set of 39,161 internal medicine admissions to Shaare Zedek Medical Center, Israel (2016-2019). Odds Ratio and Standard Error are presented. Significant results are marked.*

| Variable | **In Hospital Mortality** | | **Readmission in 30 days** | | **Increased Care** | | **LoS (log)** | |
| --- | --- | --- | --- | --- | --- | --- | --- | --- |
|  | **OR***^1,2^* | **95% CI***^2^* | **OR***^1,2^* | **95% CI***^2^* | **OR***^1,2^* | **95% CI***^2^* | **Beta***^1^* | **95% CI***^2^* |
| **Age** |  |  |  |  |  |  |  |  |
| *18-69* | — | — | — | — | — | — | — | — |
| *70-79* | 1.46*** | 1.25, 1.71 | 1.03 | 0.95, 1.12 | 0.94 | 0.86, 1.03 | -0.02* | -0.03, 0.00 |
| *>80* | 1.86*** | 1.62, 2.15 | 1.02 | 0.94, 1.11 | 0.69*** | 0.63, 0.76 | -0.08*** | -0.09, -0.06 |
| **Sex** |  |  |  |  |  |  |  |  |
| *Female* | — | — | — | — | — | — | — | — |
| *Male* | 1.02 | 0.92, 1.12 | 0.98 | 0.93, 1.05 | 1.02 | 0.95, 1.10 | 0.01* | 0.00, 0.03 |
| **Heart rate** |  |  |  |  |  |  |  |  |
| *<100* | — | — | — | — | — | — | — | — |
| *100-119* | 1.26*** | 1.13, 1.41 | 1.09* | 1.01, 1.18 | 1.11* | 1.02, 1.21 | 0.03*** | 0.02, 0.05 |
| *>120* | 1.49*** | 1.27, 1.74 | 1.12* | 1.00, 1.25 | 1.40*** | 1.24, 1.57 | 0.08*** | 0.06, 0.10 |
| **Systolic Blood Pressure** |  |  |  |  |  |  |  |  |
| *>80* | — | — | — | — | — | — | — | — |
| *<80* | 2.14*** | 1.74, 2.62 | 0.82 | 0.66, 1.00 | 2.32*** | 1.93, 2.78 | -0.15*** | -0.20, -0.11 |
| **Temperature** |  |  |  |  |  |  |  |  |
| *35-39.9* | — | — | — | — | — | — | — | — |
| *<35* | 1.94* | 1.07, 3.45 | 1.18 | 0.63, 2.06 | 2.44** | 1.42, 4.14 | 0.07 | -0.06, 0.19 |
| *>40* | 1.61 | 0.76, 3.20 | 0.92 | 0.43, 1.78 | 1.34 | 0.69, 2.50 | -0.10 | -0.25, 0.05 |
| **Blood Saturation** |  |  |  |  |  |  |  |  |
| *>80* | — | — | — | — | — | — | — | — |
| *<80* | 1.78*** | 1.41, 2.23 | 0.94 | 0.77, 1.14 | 2.83*** | 2.39, 3.35 | 0.13*** | 0.09, 0.17 |
| **1^st^ Creatinine** |  |  |  |  |  |  |  |  |
| *<1.2* | — | — | — | — | — | — | — | — |
| *1.2-1.79* | 1.30*** | 1.15, 1.47 | 1.15*** | 1.06, 1.24 | 1.05 | 0.96, 1.14 | 0.03*** | 0.01, 0.04 |
| *>1.8* | 1.91*** | 1.69, 2.15 | 1.29*** | 1.18, 1.40 | 1.14** | 1.04, 1.25 | 0.03*** | 0.01, 0.05 |
| 1^st^ **Albumin** |  |  |  |  |  |  |  |  |
| *>3.5* | — | — | — | — | — | — | — | — |
| *<3* | 1.89*** | 1.67, 2.13 | 1.33*** | 1.22, 1.45 | 1.79*** | 1.63, 1.97 | 0.56*** | 0.55, 0.58 |
| *3-3.49* | 0.96 | 0.84, 1.09 | 1.38*** | 1.28, 1.49 | 1.44*** | 1.32, 1.56 | 0.43*** | 0.42, 0.45 |
| **1^st^ Hematocrit** |  |  |  |  |  |  |  |  |
| *30-49.9* | — | — | — | — | — | — | — | — |
| *<30* | 1.36*** | 1.22, 1.52 | 1.33*** | 1.23, 1.44 | 0.93 | 0.85, 1.02 | 0.06*** | 0.05, 0.08 |
| *>50* | 1.14 | 0.79, 1.61 | 0.80 | 0.61, 1.03 | 2.09*** | 1.68, 2.59 | 0.10*** | 0.05, 0.15 |
| **1^st^ CRP** |  |  |  |  |  |  |  |  |
| *<5* | — | — | — | — | — | — | — | — |
| *5-19.9* | 1.36*** | 1.22, 1.51 | 0.96 | 0.89, 1.03 | 1.26*** | 1.16, 1.36 | 0.14*** | 0.12, 0.15 |
| *20-30* | 1.81*** | 1.54, 2.12 | 0.85* | 0.74, 0.97 | 1.55*** | 1.35, 1.76 | 0.22*** | 0.19, 0.24 |
| *>30* | 3.39*** | 2.65, 4.33 | 0.67** | 0.52, 0.85 | 2.46*** | 2.00, 3.01 | 0.28*** | 0.23, 0.33 |
| **1^st^ Sodium** |  |  |  |  |  |  |  |  |
| *130-142.9* | — | — | — | — | — | — | — | — |
| *<130* | 1.26** | 1.09, 1.46 | 1.00 | 0.90, 1.12 | 0.89 | 0.78, 1.01 | 0.00 | -0.02, 0.02 |
| *>143* | 1.46*** | 1.27, 1.67 | 1.12* | 1.01, 1.25 | 0.93 | 0.83, 1.05 | 0.03** | 0.01, 0.05 |
| **1^st^ Potassium** |  |  |  |  |  |  |  |  |
| *<5* | — | — | — | — | — | — | — | — |
| *>5* | 1.47*** | 1.30, 1.66 | 1.04 | 0.95, 1.14 | 1.37*** | 1.24, 1.51 | -0.01 | -0.03, 0.01 |
| **1^st^ Glucose** |  |  |  |  |  |  |  |  |
| *70-129.9* | — | — | — | — | — | — | — | — |
| *<70* | 2.09*** | 1.56, 2.79 | 0.92 | 0.71, 1.18 | 1.41** | 1.09, 1.81 | -0.05* | -0.10, 0.00 |
| *130-199.9* | 1.15* | 1.03, 1.28 | 1.05 | 0.98, 1.13 | 1.28*** | 1.18, 1.38 | 0.04*** | 0.03, 0.06 |
| *>200* | 1.56*** | 1.37, 1.76 | 1.10* | 1.01, 1.19 | 1.59*** | 1.45, 1.74 | 0.06*** | 0.04, 0.08 |
| **Elective Admission** |  |  |  |  |  |  |  |  |
| *0* | — | — | — | — | — | — | — | — |
| *1* | 1.21 | 0.95, 1.54 | 0.68*** | 0.60, 0.76 | 2.41*** | 2.16, 2.70 | 0.02 | 0.00, 0.04 |
| **Time Past Last Hospitalizaion** |  |  |  |  |  |  |  |  |
| *90 + OR Never* | — | — | — | — | — | — | — | — |
| *<30* | 1.23*** | 1.09, 1.39 | 1.62*** | 1.50, 1.75 | 1.04 | 0.95, 1.15 | 0.04*** | 0.03, 0.06 |
| *30-89* | 1.03 | 0.89, 1.20 | 1.34*** | 1.21, 1.48 | 1.17** | 1.04, 1.30 | 0.01 | -0.01, 0.03 |
| **Num of Prior Hospitalizaion** |  |  |  |  |  |  |  |  |
| *<3* | — | — | — | — | — | — | — | — |
| *+3* | 0.88* | 0.79, 0.98 | 1.27*** | 1.18, 1.36 | 0.82*** | 0.76, 0.89 | -0.02* | -0.03, 0.00 |
| **1^st^ Norton-Scale Physical** |  |  |  |  |  |  |  |  |
| *4* | — | — | — | — | — | — | — | — |
| *1* | 2.55*** | 1.99, 3.28 | 0.90 | 0.69, 1.16 | 1.31* | 1.03, 1.65 | 0.03 | -0.02, 0.09 |
| *2* | 1.54*** | 1.30, 1.82 | 1.18** | 1.05, 1.34 | 1.24** | 1.08, 1.41 | 0.13*** | 0.11, 0.16 |
| *3* | 1.17* | 1.01, 1.34 | 1.18*** | 1.09, 1.27 | 1.09 | 1.00, 1.20 | 0.11*** | 0.09, 0.12 |
| **1^st^ Norton-Scale Mental** |  |  |  |  |  |  |  |  |
| *4* | — | — | — | — | — | — | — | — |
| *1* | 5.40*** | 4.05, 7.21 | 0.45*** | 0.30, 0.64 | 11.0*** | 8.21, 14.7 | 0.07* | 0.01, 0.13 |
| *2* | 1.92*** | 1.60, 2.31 | 0.78** | 0.67, 0.91 | 1.61*** | 1.37, 1.88 | 0.02 | -0.01, 0.06 |
| *3* | 1.24*** | 1.09, 1.41 | 0.95 | 0.87, 1.04 | 0.97 | 0.88, 1.07 | -0.02* | -0.04, 0.00 |
| **1^st^ Norton-Scale Mobility** |  |  |  |  |  |  |  |  |
| *4* | — | — | — | — | — | — | — | — |
| *1* | 1.70* | 1.10, 2.61 | 0.98 | 0.75, 1.27 | 1.77*** | 1.32, 2.39 | 0.13*** | 0.07, 0.18 |
| *2* | 1.40 | 0.95, 2.06 | 1.15 | 0.93, 1.42 | 1.27 | 1.00, 1.62 | 0.12*** | 0.07, 0.16 |
| *3* | 1.05 | 0.76, 1.45 | 1.21* | 1.04, 1.40 | 1.21* | 1.02, 1.44 | 0.08*** | 0.05, 0.11 |
| **1^st^ Norton-Scale Activity** |  |  |  |  |  |  |  |  |
| *4* | — | — | — | — | — | — | — | — |
| *1* | 2.30*** | 1.50, 3.56 | 1.30 | 0.99, 1.69 | 1.15 | 0.85, 1.55 | 0.06 | 0.00, 0.11 |
| *2* | 2.11*** | 1.44, 3.13 | 1.01 | 0.82, 1.26 | 1.12 | 0.88, 1.44 | 0.07** | 0.03, 0.12 |
| *3* | 1.91*** | 1.39, 2.64 | 1.01 | 0.87, 1.17 | 1.04 | 0.87, 1.24 | 0.07*** | 0.04, 0.10 |
| **1^st^ Norton-Scale Incontinent** |  |  |  |  |  |  |  |  |
| *4* | — | — | — | — | — | — | — | — |
| *1* | 1.11 | 0.91, 1.36 | 1.02 | 0.89, 1.18 | 0.95 | 0.81, 1.12 | -0.04* | -0.07, -0.01 |
| *2* | 1.10 | 0.84, 1.41 | 1.10 | 0.91, 1.31 | 1.22* | 1.00, 1.49 | -0.02 | -0.06, 0.02 |
| *3* | 1.00 | 0.85, 1.19 | 1.02 | 0.92, 1.14 | 1.01 | 0.89, 1.14 | 0.01 | -0.01, 0.03 |
| **Elixhauser Diagnosis Groups** |  |  |  |  |  |  |  |  |
| ** Congestive heart failure* | 1.25*** | 1.12, 1.40 | 1.18*** | 1.10, 1.27 | 1.77*** | 1.63, 1.91 | 0.13*** | 0.12, 0.15 |
| ** Cardiac arrhythmias* | 1.03 | 0.80, 1.30 | 0.90 | 0.77, 1.05 | 1.17* | 1.01, 1.36 | 0.07*** | 0.04, 0.10 |
| ** Valvular disease* | 1.07 | 0.93, 1.23 | 1.07 | 0.97, 1.17 | 1.79*** | 1.63, 1.95 | 0.15*** | 0.14, 0.17 |
| ** Pulmonary circulation disorders* | 1.23* | 1.04, 1.45 | 1.12* | 1.01, 1.25 | 1.29*** | 1.15, 1.43 | 0.06*** | 0.03, 0.08 |
| ** Peripheral vascular disorders* | 1.04 | 0.93, 1.16 | 1.05 | 0.98, 1.13 | 1.11** | 1.03, 1.20 | 0.04*** | 0.03, 0.06 |
| ** Hypertension* | 0.86** | 0.78, 0.94 | 0.98 | 0.92, 1.04 | 1.07* | 1.00, 1.15 | 0.07*** | 0.06, 0.08 |
| ** Paralysis* | 0.81 | 0.59, 1.09 | 0.92 | 0.73, 1.15 | 0.84 | 0.65, 1.07 | 0.09*** | 0.04, 0.14 |
| **Other neurological disorders* | 1.04 | 0.88, 1.23 | 1.02 | 0.90, 1.17 | 1.31*** | 1.14, 1.50 | 0.08*** | 0.06, 0.11 |
| ** Chronic pulmonary disease* | 1.0 | 0.86, 1.14 | 1.23*** | 1.13, 1.34 | 1.59*** | 1.46, 1.74 | 0.02 | 0.00, 0.04 |
| ** Diabetes, uncomplicated* | 0.84* | 0.73, 0.97 | 0.97 | 0.89, 1.06 | 0.95 | 0.86, 1.04 | 0.02 | 0.00, 0.04 |
| ** Diabetes, complicated* | 0.60* | 0.39, 0.90 | 1.04 | 0.82, 1.30 | 0.67** | 0.51, 0.87 | 0.03 | -0.02, 0.08 |
| ** Hypothyroidism* | 1.06 | 0.94, 1.20 | 1.04 | 0.95, 1.13 | 1.00 | 0.91, 1.10 | 0.01 | 0.00, 0.03 |
| ** Renal failure* | 1.08 | 0.84, 1.37 | 0.87 | 0.72, 1.04 | 1.28** | 1.06, 1.54 | 0.02 | -0.02, 0.06 |
| ** Liver disease* | 1.62*** | 1.25, 2.08 | 1.02 | 0.84, 1.23 | 1.10 | 0.89, 1.34 | 0.02 | -0.02, 0.06 |
| ** Peptic ulcer disease* | 0.80 | 0.49, 1.25 | 1.17 | 0.88, 1.52 | 0.74 | 0.52, 1.04 | -0.02 | -0.08, 0.04 |
| ** AIDS* | 7.02 | 0.35, 43.8 | 0.66 | 0.04, 3.53 | 6.40** | 1.52, 23.2 | 0.18 | -0.14, 0.50 |
| ** Lymphoma* | 1.54* | 1.02, 2.26 | 1.15 | 0.86, 1.53 | 0.70 | 0.48, 1.01 | 0.14*** | 0.08, 0.21 |
| ** Metastatic cancer* | 2.66*** | 2.15, 3.29 | 1.10 | 0.92, 1.31 | 0.80 | 0.62, 1.02 | 0.08*** | 0.04, 0.12 |
| ** Solid tumor no metastasis* | 2.14*** | 1.87, 2.44 | 1.30*** | 1.17, 1.43 | 0.74*** | 0.64, 0.84 | 0.07*** | 0.05, 0.10 |
| ** Rheumatoid arthritis/ collagen vascular disease* | 1.08 | 0.80, 1.43 | 0.96 | 0.79, 1.16 | 1.13 | 0.91, 1.39 | 0.02 | -0.02, 0.06 |
| ** Coagulopathy* | 2.30*** | 1.83, 2.87 | 0.97 | 0.80, 1.17 | 1.20 | 0.98, 1.47 | 0.18*** | 0.14, 0.22 |
| ** Obesity* | 0.75* | 0.59, 0.95 | 0.93 | 0.82, 1.06 | 1.32*** | 1.17, 1.50 | 0.06*** | 0.04, 0.09 |
| ** Weight loss* | 0.83 | 0.54, 1.25 | 1.25 | 0.89, 1.73 | 0.71 | 0.47, 1.06 | 0.23*** | 0.15, 0.30 |
| ** Fluid & electrolyte disorder* | 1.20** | 1.07, 1.35 | 1.04 | 0.95, 1.13 | 1.72*** | 1.58, 1.89 | 0.05*** | 0.03, 0.07 |
| ** Deficiency anemias* | 0.73*** | 0.62, 0.85 | 0.88* | 0.79, 0.97 | 0.94 | 0.83, 1.06 | 0.07*** | 0.05, 0.09 |
| ** Alcohol abuse* | 0.59 | 0.22, 1.31 | 1.18 | 0.75, 1.80 | 1.35 | 0.84, 2.11 | 0.11* | 0.02, 0.20 |
| ** Drug abuse* | 0.72 | 0.10, 2.96 | 0.86 | 0.35, 1.81 | 1.68 | 0.73, 3.52 | 0.12 | -0.04, 0.27 |
| ** Psychoses* | 0.64** | 0.46, 0.86 | 1.37*** | 1.16, 1.62 | 1.09 | 0.90, 1.32 | 0.07*** | 0.04, 0.11 |
| ** Depression* | 0.74** | 0.61, 0.91 | 1.00 | 0.87, 1.14 | 0.68*** | 0.59, 0.79 | -0.01 | -0.04, 0.02 |
| *^1^* *p<0.05; **p<0.01; ***p<0.001 | | | | | | | | |
| *^2^* OR = Odds Ratio, CI = Confidence Interval | | | | | | | | |

**eTable3: Full Results of Hosmer-Lemeshow tests:**

**eTable**3: Hosmer-Lemeshow test of model calibration for in-hospital mortality (a), increased level of care (b), and 30-day readmission (c).

| **(a)** Mortality - Hosmer-Lemeshow Test*^1^* | | | |
| --- | --- | --- | --- |
| **Risk Group** | **Group Size** | **Observed***^2^* | **Expected***^2^* |
| 1 | 3747 | 5 ( 0.13% ) | 15.78  ( 0.42% ) |
| 2 | 3747 | 5 ( 0.13% ) | 21.56  ( 0.58% ) |
| 3 | 3748 | 10 ( 0.27% ) | 29  ( 0.77% ) |
| 4 | 3747 | 35 ( 0.93% ) | 41.33  ( 1.10% ) |
| 5 | 3747 | 54 ( 1.44% ) | 64.74  ( 1.73% ) |
| 6 | 3747 | 91 ( 2.43% ) | 104.22  ( 2.78% ) |
| 7 | 3747 | 190 ( 5.07% ) | 171.55  ( 4.58% ) |
| 8 | 3747 | 321 ( 8.57% ) | 287.55  ( 7.67% ) |
| 9 | 3747 | 589  ( 15.72% ) | 523.85  ( 13.98% ) |
| 10 | 3747 | 1386  ( 36.99% ) | 1426.43  ( 38.07% ) |
| *^1^* Statistic = 54.82 ; df = 8 ; P.value = 4.776794e-09 | | | |
| *^2^* Count ( % ) | | | |

| **(b)** Increased Care - Hosmer-Lemeshow Test*^1^* | | | |
| --- | --- | --- | --- |
| **Risk Group** | **Group Size** | **Observed***^2^* | **Expected***^2^* |
| 1 | 3766 | 91 ( 2.42% ) | 122.51  ( 3.25% ) |
| 2 | 3747 | 143 ( 3.82% ) | 158.32  ( 4.23% ) |
| 3 | 3747 | 158 ( 4.22% ) | 200.86  ( 5.36% ) |
| 4 | 3747 | 253 ( 6.75% ) | 252.96  ( 6.75% ) |
| 5 | 3747 | 309 ( 8.25% ) | 312.76  ( 8.35% ) |
| 6 | 3747 | 384  ( 10.25% ) | 382.63  ( 10.21% ) |
| 7 | 3747 | 530  ( 14.14% ) | 484.51  ( 12.93% ) |
| 8 | 3747 | 724  ( 19.32% ) | 631.27  ( 16.85% ) |
| 9 | 3747 | 937  ( 25.01% ) | 897.75  ( 23.96% ) |
| 10 | 3729 | 1619  ( 43.42% ) | 1704.43  ( 45.71% ) |
| *^1^* Statistic = 51.07 ; df = 8 ; P.value = 2.540666e-08 | | | |
| *^2^* Count ( % ) | | | |

| **(C)** Readmission in 30 days - Hosmer-Lemeshow Test*^1^* | | | |
| --- | --- | --- | --- |
| **Risk Group** | **Group Size** | **Observed***^2^* | **Expected***^2^* |
| 1 | 3738 | 194 ( 5.19% ) | 232.46  ( 6.2% ) |
| 2 | 3737 | 212 ( 5.67% ) | 298.76  ( 8.0% ) |
| 3 | 3750 | 327 ( 8.72% ) | 347.83  ( 9.3% ) |
| 4 | 3747 | 426 ( 11.37% ) | 407.21  ( 10.9% ) |
| 5 | 3747 | 536 ( 14.30% ) | 478.68  ( 12.8% ) |
| 6 | 3747 | 637 ( 17.00% ) | 559.99  ( 14.9% ) |
| 7 | 3747 | 688 ( 18.36% ) | 652.41  ( 17.4% ) |
| 8 | 3747 | 838 ( 22.36% ) | 769.01  ( 20.5% ) |
| 9 | 3747 | 925 ( 24.69% ) | 936.09 ( 25.0% ) |
| 10 | 3747 | 1167 ( 31.14% ) | 1267.6  ( 33.8% ) |
| 11 | 17 | 9 ( 52.94% ) | 8.94  ( 52.6% ) |
| *^1^* Statistic = 79.21 ; df = 9 ; P.value = 2.313705e-13 | | | |
| *^2^* Count ( % ) | | | |

**eTable4: Multivariable Regression Expanded Model (without Norton Scale)**

eTable4 – Multivariable regression results for In-hospital Mortality, Escalation of Care, Readmission in 30 days, and LoS without Norton Scale. Derived from derivation set of 39,161 internal medicine admissions to Shaare Zedek Medical Center, Israel (2016-2019). Odds Ratio and Standard Error are presented. Significant results are marked.

| **Variable** | **In Hospital Mortality** | | **Readmission in 30 days** | | **Increased Care** | | **LoS (log)** | |  |
| --- | --- | --- | --- | --- | --- | --- | --- | --- | --- |
|  | **OR***^1,2^* | **95% CI***^2^* | **OR***^1,2^* | **95% CI***^2^* | **OR***^1,2^* | **95% CI***^2^* | **OR***^1,2^* | **95% CI***^2^* |  |
| **Age** |  |  |  |  |  |  |  |  |  |
| *18-69* | — | — | — | — | — | — | — | — |  |
| *70-79* | 1.88*** | 1.62, 2.17 | 1.09 | 1.00, 1.18 | 1.05 | 0.96, 1.15 | 0.02* | 0.00, 0.03 | |
| *>80* | 3.18*** | 2.79, 3.63 | 1.12** | 1.04, 1.21 | 0.90* | 0.83, 0.98 | 0.01 | -0.01, 0.02 | |
| **Sex** |  |  |  |  |  |  |  |  | |
| *Female* | — | — | — | — | — | — | — | — | |
| *Male* | 0.92 | 0.84, 1.01 | 0.97 | 0.91, 1.03 | 0.98 | 0.92, 1.05 | 0.00 | -0.01, 0.01 | |
| Heart rate |  |  |  |  |  |  |  |  | |
| *<100* | — | — | — | — | — | — | — | — | |
| *100-119* | 1.33*** | 1.19, 1.48 | 1.09* | 1.01, 1.18 | 1.13** | 1.04, 1.23 | 0.04*** | 0.02, 0.05 | |
| *>120* | 1.66*** | 1.43, 1.93 | 1.12* | 1.00, 1.25 | 1.47*** | 1.31, 1.64 | 0.08*** | 0.06, 0.11 | |
| Systolic Blood Pressure |  |  |  |  |  |  |  |  | |
| *>80* | — | — | — | — | — | — | — | — | |
| *<80* | 3.13*** | 2.58, 3.78 | 0.78* | 0.63, 0.95 | 3.09*** | 2.60, 3.67 | -0.13*** | -0.17, -0.08 | |
| Temperature |  |  |  |  |  |  |  |  | |
| *35-39.9* | — | — | — | — | — | — | — | — | |
| *<35* | 4.60*** | 2.65, 7.84 | 1.06 | 0.57, 1.84 | 4.76*** | 2.92, 7.70 | 0.13* | 0.00, 0.26 | |
| *>40* | 2.22* | 1.06, 4.40 | 0.94 | 0.44, 1.82 | 1.61 | 0.83, 2.99 | -0.06 | -0.21, 0.10 | |
| **Blood Oxygen Saturation** |  |  |  |  |  |  |  |  | |
| *>80* | — | — | — | — | — | — | — | — | |
| *<80* | 1.91*** | 1.53, 2.38 | 0.95 | 0.78, 1.15 | 2.92*** | 2.47, 3.44 | 0.15*** | 0.11, 0.19 | |
| 1^st^ **Creatinine** |  |  |  |  |  |  |  |  | |
| *<1.2* | — | — | — | — | — | — | — | — | |
| *1.2-1.79* | 1.24*** | 1.10, 1.39 | 1.16*** | 1.07, 1.25 | 1.03 | 0.95, 1.12 | 0.03*** | 0.02, 0.05 | |
| *>1.8* | 1.83*** | 1.63, 2.06 | 1.31*** | 1.20, 1.42 | 1.14** | 1.03, 1.25 | 0.05*** | 0.03, 0.07 | |
| 1^st^ **Albumin** |  |  |  |  |  |  |  |  | |
| *>3.5* | — | — | — | — | — | — | — | — | |
| *<3* | 2.92*** | 2.61, 3.28 | 1.42*** | 1.31, 1.54 | 2.25*** | 2.05, 2.46 | 0.63*** | 0.61, 0.65 | |
| *3-3.49* | 1.20** | 1.06, 1.36 | 1.46*** | 1.36, 1.57 | 1.57*** | 1.45, 1.71 | 0.48*** | 0.46, 0.49 | |
| 1^st^ Hematocrit |  |  |  |  |  |  |  |  | |
| *30-49.9* | — | — | — | — | — | — | — | — | |
| *<30* | 1.38*** | 1.24, 1.54 | 1.34*** | 1.24, 1.45 | 0.94 | 0.86, 1.03 | 0.07*** | 0.05, 0.09 | |
| *>50* | 1.37 | 0.96, 1.91 | 0.79 | 0.60, 1.02 | 2.20*** | 1.77, 2.72 | 0.10*** | 0.05, 0.15 | |
| 1^st^ CRP |  |  |  |  |  |  |  |  | |
| *<5* | — | — | — | — | — | — | — | — | |
| *5-19.9* | 1.44*** | 1.30, 1.59 | 0.97 | 0.90, 1.04 | 1.29*** | 1.19, 1.40 | 0.15*** | 0.13, 0.16 | |
| *20-30* | 1.98*** | 1.70, 2.31 | 0.85* | 0.75, 0.97 | 1.60*** | 1.41, 1.82 | 0.23*** | 0.20, 0.25 | |
| *>30* | 3.44*** | 2.72, 4.34 | 0.67** | 0.52, 0.85 | 2.51*** | 2.05, 3.06 | 0.29*** | 0.24, 0.33 | |
| 1^st^ **Sodium** |  |  |  |  |  |  |  |  | |
| *130-142.9* | — | — | — | — | — | — | — | — | |
| *<130* | 1.21** | 1.05, 1.40 | 1.02 | 0.91, 1.13 | 0.86* | 0.76, 0.98 | 0.01 | -0.02, 0.03 | |
| *>143* | 2.06*** | 1.81, 2.35 | 1.12* | 1.01, 1.25 | 1.15* | 1.03, 1.29 | 0.05*** | 0.02, 0.07 | |
| 1^st^ Potassium |  |  |  |  |  |  |  |  | |
| *<5* | — | — | — | — | — | — | — | — | |
| *>5* | 1.65*** | 1.46, 1.85 | 1.04 | 0.94, 1.14 | 1.46*** | 1.33, 1.61 | 0.00 | -0.02, 0.02 | |
| 1^st^ Glucose |  |  |  |  |  |  |  |  | |
| *70-129.9* | — | — | — | — | — | — | — | — | |
| *<70* | 2.36*** | 1.78, 3.11 | 0.92 | 0.71, 1.18 | 1.50** | 1.17, 1.92 | -0.04 | -0.09, 0.01 | |
| *130-199.9* | 1.26*** | 1.14, 1.40 | 1.06 | 0.99, 1.13 | 1.34*** | 1.24, 1.45 | 0.05*** | 0.04, 0.07 | |
| *>200* | 1.93*** | 1.71, 2.17 | 1.09* | 1.00, 1.19 | 1.79*** | 1.64, 1.96 | 0.08*** | 0.06, 0.09 | |
| Elective Admission |  |  |  |  |  |  |  |  | |
| *0* | — | — | — | — | — | — | — | — | |
| *1* | 0.90 | 0.71, 1.12 | 0.65*** | 0.57, 0.73 | 2.20*** | 1.97, 2.45 | -0.01 | -0.03, 0.01 | |
| Time Since Last Hospitalization | |  |  |  |  |  |  |  | |
| *90 + OR Never* | — | — | — | — | — | — | — | — | |
| *<30* | 1.30*** | 1.15, 1.46 | 1.64*** | 1.51, 1.77 | 1.07 | 0.97, 1.17 | 0.06*** | 0.04, 0.07 | |
| *30-89* | 1.09 | 0.94, 1.26 | 1.36*** | 1.23, 1.49 | 1.18** | 1.06, 1.32 | 0.02 | 0.00, 0.04 | |
| Number of Prior Hospitalizations | |  |  |  |  |  |  |  | |
| *<3* | — | — | — | — | — | — | — | — | |
| *+3* | 0.99 | 0.89, 1.10 | 1.30*** | 1.21, 1.39 | 0.86*** | 0.80, 0.93 | 0.00 | -0.02, 0.01 | |
| **Elixhauser Diagnosis Groups** | |  |  |  |  |  |  |  | |
| *Congestive heart failure* | 1.28*** | 1.15, 1.43 | 1.20*** | 1.11, 1.29 | 1.78*** | 1.65, 1.93 | 0.15*** | 0.13, 0.16 | |
| *Cardiac arrhythmias* | 0.91 | 0.72, 1.15 | 0.89 | 0.77, 1.04 | 1.13 | 0.97, 1.31 | 0.06*** | 0.03, 0.09 | |
| *Valvular disease* | 0.99 | 0.87, 1.13 | 1.06 | 0.97, 1.16 | 1.68*** | 1.54, 1.83 | 0.15*** | 0.13, 0.17 | |
| *Pulmonary circulation disorders* | 1.10 | 0.94, 1.29 | 1.13* | 1.01, 1.25 | 1.23*** | 1.11, 1.37 | 0.06*** | 0.04, 0.08 | |
| *Peripheral vascular disorders* | 1.04 | 0.93, 1.15 | 1.06 | 0.99, 1.14 | 1.11** | 1.02, 1.19 | 0.05*** | 0.03, 0.06 | |
| *Hypertension* | 0.84*** | 0.76, 0.92 | 0.98 | 0.92, 1.04 | 1.06 | 0.99, 1.14 | 0.07*** | 0.06, 0.08 | |
| *Paralysis* | 1.27 | 0.92, 1.72 | 1.01 | 0.80, 1.26 | 1.12 | 0.88, 1.41 | 0.16*** | 0.12, 0.21 | |
| *Other neurological disorders* | 1.66*** | 1.41, 1.95 | 1.05 | 0.92, 1.20 | 1.76*** | 1.54, 2.00 | 0.14*** | 0.11, 0.17 | |
| *Chronic pulmonary disease* | 0.95 | 0.83, 1.08 | 1.24*** | 1.14, 1.35 | 1.54*** | 1.41, 1.67 | 0.02** | 0.01, 0.04 | |
| *Diabetes, uncomplicated* | 0.81** | 0.71, 0.93 | 0.98 | 0.90, 1.07 | 0.93 | 0.85, 1.02 | 0.02* | 0.00, 0.04 | |
| *Diabetes, complicated* | 0.59** | 0.39, 0.86 | 1.06 | 0.84, 1.32 | 0.65** | 0.49, 0.84 | 0.03 | -0.02, 0.09 | |
| *Hypothyroidism* | 1.11 | 0.98, 1.25 | 1.04 | 0.96, 1.14 | 1.02 | 0.92, 1.12 | 0.02* | 0.00, 0.04 | |
| *Renal failure* | 1.08 | 0.85, 1.35 | 0.87 | 0.72, 1.04 | 1.25* | 1.04, 1.50 | 0.02 | -0.02, 0.06 | |
| *Liver disease* | 1.61*** | 1.25, 2.05 | 1.04 | 0.85, 1.25 | 1.09 | 0.89, 1.33 | 0.03 | -0.01, 0.07 | |
| *Peptic ulcer disease* | 0.80 | 0.50, 1.24 | 1.17 | 0.89, 1.53 | 0.76 | 0.53, 1.05 | -0.02 | -0.07, 0.04 | |
| *AIDS* | 4.41 | 0.23, 26.1 | 0.65 | 0.04, 3.47 | 5.54* | 1.32, 20.1 | 0.16 | -0.17, 0.49 | |
| *Lymphoma* | 1.30 | 0.88, 1.88 | 1.17 | 0.87, 1.55 | 0.67* | 0.46, 0.96 | 0.16*** | 0.10, 0.22 | |
| *Metastatic cancer* | 2.77*** | 2.25, 3.39 | 1.14 | 0.95, 1.36 | 0.86 | 0.67, 1.08 | 0.12*** | 0.08, 0.16 | |
| *Solid tumor no metastasis* | 2.00*** | 1.75, 2.27 | 1.32*** | 1.19, 1.46 | 0.72*** | 0.64, 0.82 | 0.09*** | 0.07, 0.11 | |
| *Rheumatoid arthritis/  collagen vascular disease* | 1.05 | 0.79, 1.38 | 0.97 | 0.80, 1.17 | 1.11 | 0.90, 1.36 | 0.02 | -0.02, 0.06 | |
| *Coagulopathy* | 2.33*** | 1.87, 2.89 | 0.98 | 0.80, 1.18 | 1.21 | 0.99, 1.48 | 0.19*** | 0.15, 0.23 | |
| *Obesity* | 0.74** | 0.59, 0.92 | 0.94 | 0.83, 1.07 | 1.30*** | 1.15, 1.46 | 0.07*** | 0.04, 0.09 | |
| *Weight loss* | 0.92 | 0.60, 1.39 | 1.31 | 0.93, 1.81 | 0.74 | 0.49, 1.09 | 0.25*** | 0.17, 0.33 | |
| *Fluid & electrolyte disorder* | 1.32*** | 1.18, 1.47 | 1.05 | 0.97, 1.15 | 1.81*** | 1.66, 1.98 | 0.06*** | 0.04, 0.08 | |
| *Deficiency anemias* | 0.74*** | 0.64, 0.86 | 0.88* | 0.79, 0.98 | 0.94 | 0.83, 1.06 | 0.07*** | 0.05, 0.10 | |
| *Alcohol abuse* | 0.63 | 0.25, 1.36 | 1.16 | 0.74, 1.76 | 1.43 | 0.90, 2.20 | 0.11* | 0.02, 0.21 | |
| *Drug abuse* | 1.19 | 0.19, 4.16 | 0.83 | 0.34, 1.75 | 1.97 | 0.92, 3.94 | 0.15 | -0.01, 0.30 | |
| *Psychoses* | 0.81 | 0.59, 1.08 | 1.43*** | 1.21, 1.68 | 1.22* | 1.00, 1.46 | 0.11*** | 0.08, 0.15 | |
| *Depression* | 0.83 | 0.68, 1.01 | 1.01 | 0.88, 1.15 | 0.73*** | 0.63, 0.84 | 0.01 | -0.02, 0.04 | |
| ^1^ *p<0.05; **p<0.01 | | | | | | | | |  |
| *^2^* OR = Odds Ratio, CI = Confidence Interval | | | | | | | | |  |
